# Cell Stress and Cell Death

**DOI:** 10.1155/2010/245803

**Published:** 2010-03-22

**Authors:** Afshin Samali, Simone Fulda, Adrienne M. Gorman, Osamu Hori, Srinivasa M. Srinivasula

**Affiliations:** ^1^Apoptosis Research Centre, School of Natural Sciences, National University of Ireland, Galway, Ireland; ^2^Children's Hospital, Ulm University, Eythstrasse. 24, 89075 Ulm, Germany; ^3^Department of Neuroanatomy, Kanazawa University Graduate School of Medical Science, Kanazawa 920-8641, Japan; ^4^Laboratory of Immune Cell Biology, National Cancer Institute, National Institutes of Health, Bethesda, MD, 20892, USA

This special issue on Cell Stress and Cell Death is aimed at bringing together recent developments in the fields of cellular stress and cell death and, in particular, the interplay between cell stress responses and cell death. The special issue opens with a review by S. Fulda et al. which provides an overview of how cells can respond to stress in a variety of ways ranging from the activation of survival pathways to the initiation of cell death that eventually eliminates damaged cells. Whether cells mount a protective response or succumb to death depends to a large extent on the nature and duration of the stress as well as the cell type. For example, milder stresses can lead to protection through activation of the heat shock response or the unfolded protein response (UPR). This review also describes several types of cell death (e.g., apoptosis, necrosis, pyroptosis, or autophagic cell death) and the mechanism by which a cell dies often depends on various exogenous factors as well as the cell's ability to handle the stress to which it is exposed. The implications of cellular stress responses for human physiology and disease are multifold and are discussed in this review in the context of some major world health issues such as diabetes, Parkinson's disease, myocardial infarction, and cancer. 

There are many molecules and cellular processes that play critical roles in normal cell signaling and survival responses, while also having a dual role in inducing cell death. A number of papers in this special issue, covering endoplasmic reticulum (ER) stress and Ca^2+^, address this topic. In recent years there has been a significant increase in the number of papers in the field dealing with ER stress and ER stress-induced cell death. This reflects the growing recognition of the importance of the ER in cell stress and in different modes of cell death. The ER is the site of folding of membrane and secreted proteins in the cell. Physiological or pathological processes that disturb protein folding in the ER initiate a complex intracellular signal transduction pathway, known as the UPR. This response is an attempt to reestablish ER homeostasis, although it can also lead to cell death. The review by A. Samali et al. provides a comprehensive overview of current methodologies for monitoring the UPR and ER stress, and it puts together a set of criteria to assess this response, which will be useful for researchers who wish to examine these phenomena in different model systems. The UPR is essentially tailored to reestablish ER homeostasis and it can also signal through other adaptive mechanisms involving the stimulation of autophagy. However, when ER stress is persistent, the cytoprotective functions of the UPR and autophagy can switch to cell death-promoting mechanisms. A review by T. Verfaillie et al. discusses the relationship between ER stress and autophagy and the implications for cancer therapy. Recently, a variety of anticancer therapies have been linked to the induction of ER stress in cancer cells, envisaging strategies that stimulate prodeath function or block its prosurvival function, to improve tumoricidial action. A better understanding of the molecular mechanisms that determine the final outcome of UPR and autophagy activation by chemotherapeutic agents will offer new opportunities to improve existing cancer therapies as well as revealing novel targets for cancer treatment. Prolonged or severe ER stress has been linked to induction of apoptosis. Caspase activation is one of the key steps in commitment of cells to apoptosis and is dependent on mitochondrial outer membrane permeabilization (MOMP) and the release of cytochrome *c* from the mitochondria. The research article by S. Gupta et al. investigates the mechanism of ER stress-induced MOMP using thapsigargin as an inducer of ER stress. They genetically dissected the role of caspase-9, -3, and -2 in the induction of MOMP by ER stress using embryonic fibroblasts derived from knockout mice and also treated cells with chemical and molecular inhibitors of the mitochondrial permeability transition. Their results suggest that caspase-9 and -2, Bcl-2 family members, and the mitochondrial permeability transition pore all play a role in MOMP during ER stress-induced apoptosis. 

Ca^2+^ is an important second messenger which is also poised at the intersection between cell survival and cell death. The review by C. Cerella et al. examines the events that occur during Ca^2+^ toxicity and how reparative or death pathways can be activated. They also discuss the observations that while Ca^2+^ can elicit these opposing responses, it also plays a role as a second messenger in signal transduction associated with cell death and survival. 

Knowledge of cell stress and cell death pathways, and the interplay between these, beneficially provides us with new ways to tackle diseases, particularly cancers and degenerative disease. Resistance to apoptosis is a feature of many cancer cells and this is the subject of the review by S. Fulda which summarizes the main mechanisms by which cancer cells evade the intrinsic and extrinsic apoptosis pathways. Generally, altered ratios of key pro- and antiapoptotic proteins are responsible for resistance to cell death; for example, altered ratios of Bcl-2 family proteins as well as increased expression of caspase inhibitors (IAPs) regulate the intrinsic pathway, while reduced sensitivity of extrinsic pathways occurs due to decreased expression of death receptors on the plasma membrane and increased expression of intracellular decoy proteins. Given this increased resistance to cell death pathways, the search for novel molecules to induce cell death in cancer cells is an important goal. Histone deacetylase inhibitors have become a promising new avenue for cancer therapy, and many are currently in clinical trials for various tumor types. The research article by N. Rivera-Del Valle et al. demonstrates that a novel hydroxamic acid histone deacetylase inhibitor, PCI-24781, exerts cytotoxicity and histone alterations in leukemia cells, via a mechanism that is dependent on caspase-8 and Fas-associated death domain. Another avenue for identifying novel therapeutic targets for cancers is study of inflammation, since many human malignancies have been strongly linked with chronic inflammation. The review by C. Sobolewski et al. describes the role of cyclooxygenase-2 in these diseases. This enzyme is a member of a family, which catalyzes the rate-limiting step of prostaglandin biosynthesis. Cyclooxygenase-2 is upregulated during both inflammation and cancer, and has been described to modulate cell proliferation and apoptosis mainly in solid tumors and more recently in hematological malignancies. Thus, the use of cyclooxygenase-2 inhibitors, together with other therapeutic strategies, may further improve the efficiency of anticancer treatments in the clinic. 

In contrast to cancers, an important aim of research into degenerative diseases is to discover novel ways to *inhibit* cell death. One such disease is Parkinson's disease, where there are currently no therapies which halt the degeneration of dopaminergic neurons. In the research article by K. Mnich et al. the ability of the endogenous cannabinoid, anandamide, to inhibit apoptotic cell death by the Parkinson mimetic, 6-hydroxydopamine, was shown. The protection provided by anandamide involved activation of phosphatidylinositol 3-kinase and prevention of 6-hydroxydopamine induced activation of c-Jun-NH2-terminal kinase (JNK). These data add to the growing body of literature concerning cannabinoids and Parkinson's disease. Certain other neurodegenerative diseases, termed tauopathies, feature filamentous tau-positive protein inclusions in neurons and glia, which are characterized by the expression of stress response proteins, particularly heat shock proteins (Hsps), in these inclusions. The article by L. Schwarz et al. investigated the contribution of small Hsps, Hsp27, and *α*B-crystallin, to neurodegenerative diseases by analyzing the association of Hsp27 with pathological lesions of tauopathies. Their results suggest distinct mechanisms for Hsp27 action in glial and neuronal cells, with prominent expression in unstressed astrocytes but with low expression observed in neurons even after stress situations.

We hope that this special issue will alert researchers to some new developments in the fields of cell stress and cell death, particularly the interplay between prosurvival and prodeath responses, and how our knowledge of these can direct our efforts in discovering new therapeutic strategies for the treatment of cancers and degenerative diseases.

*Afshin Samali* *Afshin Samali* *Simone Fulda* *Simone Fulda* *Adrienne M. Gorman* *Adrienne M. Gorman* *Osamu Hori* *Osamu Hori* *Srinivasa M. Srinivasula* *Srinivasa M. Srinivasula*

